# Upregulated ZBP1 Is Associated with B-Cell Dysregulation in Systemic Lupus Erythematosus

**DOI:** 10.3390/biomedicines14020451

**Published:** 2026-02-17

**Authors:** Yiying Yang, Ke Liu, Hao Ma, Litao Lu, Ganqian Zhu, Xiaoxia Zuo, Huali Zhang, Yaxi Zhu, Muyao Guo

**Affiliations:** 1 Department of Rheumatology, Xiangya Hospital, Department of Pathophysiology, Xiangya School of Basic Medicine Science, Central South University, Changsha 410011, China; yangyy1018@csu.edu.cn (Y.Y.);; 2 Sepsis Translational Medicine Key Laboratory of Hunan Province, Changsha 410011, China; 3 National Medicine Functional Experimental Teaching Center, Central South University, Changsha 410011, China; 4 Postdoctoral Research Station of Biology, School of Basic Medicine Science, Central South University, Changsha 410011, China; 5 School of Biomedical Sciences, Hunan University, Changsha 410011, China; 6 Provincial Clinical Research Center for Rheumatic and Immunologic Diseases, Xiangya Hospital, Central South University, Changsha 410011, China; 7 National Clinical Research Center for Geriatric Disorders, Xiangya Hospital, Central South University, Changsha 410011, China

**Keywords:** SLE, B cell, ZBP1, IFN, cell cycle, plasma cell differentiation

## Abstract

**Background/Objectives:** Systemic lupus erythematosus (SLE) is an autoimmune disease characterized by B-cell hyperactivation and excessive autoantibody production. Z-DNA binding protein 1 (ZBP1), an innate immune sensor involved in nucleic acid recognition and cell death signaling, has been implicated in antiviral and inflammatory responses. However, its role in B-cell dysregulation during SLE remains unclear. **Methods:** Integrative transcriptomic analyses were performed using public datasets (GSE61635, GSE235658, GSE136035, and GSE163497) to determine the expression pattern and biological functions of ZBP1 in SLE. Bulk RNA-seq and single-cell RNA-seq data were used to evaluate *ZBP1* expression across B-cell subsets. Correlations between ZBP1 expression, disease activity, and immunological parameters were assessed. RNA-seq data following *ZBP1* knockdown were analyzed to explore its potential downstream pathways and molecular networks. In addition, in vitro *ZBP1* knockdown experiments were conducted to examine its effects on B-cell activation, plasma cell differentiation, and antibody production. **Results:** ZBP1 was significantly upregulated in peripheral blood and B cells from SLE patients and was enriched in pathways related to type I interferon signaling and cytokine-mediated immune responses. Single-cell transcriptomic profiling further revealed elevated *ZBP1* expression across multiple B-cell subsets, including naïve B cells, memory B cells, age-associated B cells (ABCs), and plasma cells. Clinically, ZBP1 expression in peripheral B cells was positively correlated with CD86 mean fluorescence intensity (MFI), SLE Disease Activity Index (SLEDAI) scores, and serum IgG levels, suggesting a link between ZBP1 and B-cell activation. RNA-seq analysis following *ZBP1* silencing demonstrated that ZBP1 regulates genes involved in the cell cycle, DNA replication, and p53 signaling, indicating its potential role in promoting B-cell proliferation and activation. Functionally, *ZBP1* silencing impaired B-cell activation, reduced plasma cell differentiation, and decreased immunoglobulin production in vitro. **Conclusions:** Our study identifies ZBP1 as a molecule upregulated in SLE B cells and associated with B-cell activation and disease activity. Although direct causality remains to be established, the data indicate that ZBP1 may contribute to SLE pathogenesis by modulating cell cycle-related pathways and promoting aberrant B-cell responses, highlighting its potential as a biomarker and a candidate therapeutic target in SLE.

## 1. Introduction

Systemic lupus erythematosus (SLE) is a prototypic autoimmune disease characterized by loss of immune tolerance, production of autoantibodies, and multi-organ inflammation [1,2]. Among various immune cells involved, B cells play a central role in the initiation and perpetuation of SLE pathogenesis through excessive activation, autoantibody secretion, and cytokine production [3,4,5,6,7,8,9]. Dysregulated B-cell activation contributes to immune complex deposition, complement activation, and tissue damage. However, the molecular mechanisms underlying aberrant B-cell activation in SLE remain incompletely understood.

Recent advances in transcriptomic profiling have revealed that type I interferon (IFN) signaling and viral response pathways are among the most prominent transcriptional signatures in SLE [10,11,12,13]. Type I IFNs can enhance B-cell survival, promote plasma cell differentiation, and amplify autoantibody production [14,15,16,17]. Several interferon-stimulated genes (ISGs) have been implicated in SLE, including IRF7, IFITM1, ISG15, RSAD2 and MX1 [18,19,20,21,22,23,24]. Nevertheless, the upstream molecular regulators that drive and sustain this interferon signature in B cells are not fully identified.

Z-DNA binding protein 1 (ZBP1), also known as DAI or DLM-1, is a cytosolic nucleic acid sensor originally identified for its ability to bind left-handed Z-DNA and Z-RNA structures [25,26]. ZBP1 functions as an innate immune adaptor that activates necroptosis and inflammasome signaling in response to viral infection, particularly through its interaction with RIPK3 and MLKL [27,28,29]. Beyond its classical antiviral role, accumulating evidence suggests that ZBP1 also contributes to sterile inflammation and autoimmune responses [30,31,32,33,34]. For instance, aberrant activation of the ZBP1–RIPK3 pathway has been linked to autoinflammatory conditions and lupus-like phenotypes in murine models. Moreover, ZBP1 has been implicated in multiple human autoimmune contexts: in autoimmune photosensitivity, ZBP1 stabilizes cytosolic Z-DNA after UVB exposure and amplifies type I interferon responses; in TNF-driven inflammatory skin disorders, ZBP1 mediates cGAS/STING-dependent interferon production and apoptosis/necroptosis; in lupus nephritis, ZBP1 regulates PANoptosis in podocytes and contributes to renal injury. In primary Sjögren’s syndrome (pSS), ZBP1 participates in STING-mediated type I interferon activation in monocytes and plasmacytoid dendritic cells [35,36], while in RA-associated lung disease [37], ZBP1 is upregulated in myeloid cells, contributing to pro-inflammatory and pro-fibrotic responses. Experimental studies in pSS models further show that therapeutic interventions targeting upstream pathways, such as baicalin and quercetin, can downregulate ZBP1 and ameliorate inflammation. Collectively, these findings indicate that ZBP1 may act as a critical amplifier of inflammatory and autoimmune processes across multiple diseases. However, its specific role in human autoimmune disorders, particularly in B-cell-mediated diseases such as SLE, remains poorly explored.

Given that viral infection-related pathways and type I IFN signaling are enriched in SLE, and ZBP1 serves as a key mediator in both processes, we hypothesized that ZBP1 might be involved in SLE pathogenesis by regulating B-cell activation and function. To address this, we performed an integrative multi-omics analysis combining bulk RNA-seq, single-cell RNA-seq, and functional transcriptomic datasets.

In the present study, we first analyzed two independent bulk transcriptomic datasets (GSE61635 and GSE235658) and found that ZBP1 expression was significantly upregulated in peripheral blood and B cells from SLE patients, accompanied by enrichment of pathways related to type I IFN and cytokine signaling. Next, single-cell RNA-seq analysis (GSE136035) revealed that ZBP1 expression was consistently elevated across multiple B-cell subsets—including naïve B cells, memory B cells, age-associated B cells (ABCs), and plasma cells—indicating a broad upregulation during B-cell differentiation. Flow cytometric analysis further confirmed increased ZBP1 protein levels in peripheral B cells of SLE patients, and ZBP1 expression correlated positively with CD86 expression, SLE disease activity index (SLEDAI), and serum IgG levels, linking ZBP1 to B-cell activation and clinical severity. Finally, transcriptomic analysis following ZBP1 knockdown (GSE163497) showed that ZBP1 regulates genes involved in the cell cycle, DNA replication, and p53 signaling, suggesting a potential mechanism through which ZBP1 promotes B-cell proliferation and activation.

Collectively, our findings reveal that ZBP1 is markedly upregulated in SLE B cells and is associated with enhanced activation and disease activity. These data indicate that ZBP1 may contribute to B-cell hyperactivation by influencing interferon and cell cycle related pathways, providing new insights into the molecular mechanisms of SLE and identifying ZBP1 as a potential biomarker and therapeutic target.

## 2. Methods and Materials

### 2.1. Bulk and Knockdown RNA-Seq Data Analysis

Transcriptomic datasets were retrieved from the Gene Expression Omnibus (GEO) database to investigate the expression and functional relevance of ZBP1 in SLE.

Two bulk RNA-seq datasets (Table 1), GSE61635 (whole blood) and GSE235658 (B cells), were analyzed to identify differentially expressed genes (DEGs) between SLE patients and healthy controls (HCs). DEG analysis was performed in R (v4.3.2) using the DESeq2 package. The thresholds for significance were set as |log_2_FC| ≥ 1 and adjusted *p*-value (FDR) < 0.05, with the Benjamini–Hochberg procedure applied to correct for multiple testing. Volcano plots were generated to visualize expression changes, and Venn diagrams were used to identify overlapping DEGs between datasets.

To further elucidate the downstream molecular mechanisms of ZBP1, we analyzed the GSE163497 dataset [39], which includes multiple myeloma (MM) cell lines transduced with short hairpin RNAs targeting ZBP1 (shZBP1) and matched scramble controls. DEGs between shZBP1 and scramble groups were determined using DESeq2 under the same significance criteria. Functional enrichment of DEGs was conducted using the clusterProfiler R package to identify significantly enriched Gene Ontology (GO) terms [40] and Kyoto Encyclopedia of Genes and Genomes (KEGG) pathways [41]. Protein–protein interaction (PPI) networks were constructed using the STRING database (https://string-db.org, accessed on 21 October 2025) [42], and key regulatory clusters were extracted using the MCODE plugin in Cytoscape (v3.10.1) to identify hub modules.

### 2.2. Single-Cell RNA-Seq Data Analysis

The single-cell RNA-seq dataset GSE136035 [43] was downloaded from the GEO database to investigate the cell-type-specific expression pattern of ZBP1 in B cells from SLE patients and HCs. All analyses were performed using the Seurat (v4.0) R package following standard workflows, as previously described [44].

(1) Data preprocessing and quality control:

Raw count matrices were loaded into Seurat, and low-quality cells with fewer than 200 detected genes or with >10% mitochondrial gene content were excluded. Genes expressed in fewer than 3 cells were also removed. The data were normalized using the LogNormalize method with a scale factor of 10,000. The top 2000 highly variable genes were selected for downstream analysis.

(2) Dimensionality reduction and clustering:

Principal component analysis (PCA) was performed to reduce data dimensionality, and the top 20 principal components were used for clustering with the FindClusters function (resolution = 0.5). Two-dimensional visualization of cell distribution was generated using Uniform Manifold Approximation and Projection (UMAP), implemented with the RunUMAP function.

(3) Cell-type annotation:

Cell clusters were annotated according to canonical marker genes and published references.

Naïve B cells: CD19^+^, CD27^−^, MS4A1^+^

Memory B cells: CD19^+^, CD27^+^, TNFRSF13B^+^

ABCs: CD19^+^, CD11c^+^, T-bet^+^ (TBX21)

Plasma cells: CD38^+^, SDC1^+^ (CD138)

Cluster identities were further confirmed by differential expression analysis using FindAllMarkers (min.pct = 0.25, logfc.threshold = 0.25).

(4) Visualization of ZBP1 expression:

The expression pattern of ZBP1 across all B-cell subsets was visualized using UMAP feature plots (FeaturePlot function), allowing spatial visualization of gene expression in the reduced dimension.

Additionally, DotPlot was employed to quantitatively display ZBP1 expression levels and the proportion of ZBP1-positive cells across distinct B-cell clusters.

### 2.3. Functional Enrichment Analysis

To elucidate the biological functions of target genes, GO and KEGG enrichment analyses were conducted using the clusterProfiler R package. GO terms were categorized into Biological Process (BP), Molecular Function (MF), and Cellular Component (CC). Enriched terms and pathways were visualized using ggplot2 and enrichplot, with *p* < 0.05 considered statistically significant.

### 2.4. PPI Network Analysis

PPI networks of DEGs were constructed using the STRING database (https://string-db.org, accessed on 21 October 2025) with a minimum interaction score of >0.7. Disconnected nodes were hidden, and the resulting networks were visualized using Cytoscape. Hub modules were identified using the MCODE algorithm (degree cutoff = 2, node score cutoff = 0.2, k-core = 2), enabling detection of functionally enriched subnetworks.

### 2.5. Human Samples

A total of 32 newly diagnosed SLE patients fulfilling the 2019 European League Against Rheumatism (EULAR)/American College of Rheumatology (ACR) classification criteria [45] and 12 age- and sex-matched HCs were recruited from the Department of Rheumatology and Immunology, Xiangya Hospital, Central South University.

All procedures were approved by the Ethics Committee of Xiangya Hospital, Central South University (approval number: 201212074), and written informed consent was obtained from all participants. Clinical characteristics of the participants are summarized in Table 2.

### 2.6. Isolation of Peripheral Blood Mononuclear Cells (PBMCs)

PBMCs were isolated from freshly collected heparinized blood using Ficoll-Paque PLUS (GE Healthcare, Chicago, IL, USA) density gradient centrifugation according to the manufacturer’s protocol. Cells were washed twice with phosphate-buffered saline (PBS), counted, and resuspended in PBS or RPMI-1640 supplemented with 10% fetal bovine serum (FBS) for downstream flow cytometric analysis.

### 2.7. B-Cell Isolation

Human CD19^+^ B cells were isolated from the PBMCs of HCs and SLE patients by using antibody-conjugated magnetic beads (Miltenyi, CD19 MicroBeads, Human, Köln, Germany), as previously described [46].

### 2.8. siRNA-Mediated ZBP1 Knockdown in B Cells

RAJI B cells were cultured in RPMI 1640 medium supplemented with 10% FBS at 37 °C in a humidified atmosphere containing 5% CO_2_. Small interfering RNA targeting ZBP1 (si-*ZBP1*, GGATGAGCAGTCCAAAGCA) and the corresponding negative control siRNA (si-NC) were transfected into RAJI B cells according to the manufacturer’s instructions using the indicated transfection reagent (Ribo Bio, Guangzhou, China), as described previously [47]. Knockdown efficiency was assessed at the mRNA level by quantitative real-time PCR prior to subsequent functional analyses.

### 2.9. Real-Time Quantitative PCR (RT-qPCR)

Total RNA was extracted from the B cells of SLE patients using TRIzol (Accurate Biology, Changsha, China), and reverse transcribed into cDNA with the Evo M-MLV RT Mix Kit (Accurate Biology, AG11728, Changsha, China). RT-qPCR analysis was performed using SYBR^®^ Green Premix (Accurate Biology, AG11701, Changsha, China) according to the manufacturer’s instructions. The PCR primers used are listed in Table 3.

### 2.10. Flow Cytometric Analysis

PBMCs were first blocked with anti-Fc reagent (BioLegend, 422302, human, San Diego, CA, USA) for 15 min on ice to prevent nonspecific binding. For surface staining, cells were incubated with fluorochrome-conjugated antibodies against CD19 (BioLegend, APC-Cy7, 302218, California, USA), CD27 (BioLegend, APC, 356410, San Diego, CA, USA), CD38 (BioLegend, PE-Cy7, 356608, California, USA), and CD86 (BioLegend, PerCP/Cy5.5, 374216, California, USA) for 30 min on ice. Through labeling of CD19, CD38, and CD27, B-cell subsets were gated as follows [46,48,49]: naïve B cells (NB, CD19^+^CD27^−^CD38^+/−^), memory B cells (MB, CD19^+^CD27^+^CD38^−^), plasma cells (PC, CD19^+^CD27^+^CD38^+/bright^), as shown in Supplementary Figure S1.

Intracellular ZBP1 staining was performed using the Fixation/Permeabilization Kit (Invitrogen, Carlsbad, CA, USA) following the manufacturer’s instructions, as previously described [47]. Cells were fixed using Fixation/Permeabilization Concentrate (eBioscience, 00–5523–00, Carlsbad, CA, USA) and Fixation/Perm Diluent (ratio 1:3) for 45 min at room temperature, washed twice with 1× Permeabilization Buffer, and incubated overnight at 4 °C with the anti-ZBP1 primary antibody (Invitrogen, PA5-143927, Carlsbad, CA, USA). After washing, cells were stained with Alexa Fluor 488–conjugated goat anti-rabbit IgG (CST, 4412, Danvers, MA, USA) for 1 h at room temperature. Finally, cells were washed and analyzed on a BD LSRFortessa™ flow cytometer (BD Biosciences, San Jose, CA, USA). Data were processed using FlowJo v10.8, and mean fluorescence intensity (MFI) was used to quantify ZBP1 expression in B-cell subsets.

### 2.11. Enzyme-Linked Immunosorbent Assay (ELISA)

Immunoglobulin levels (e.g., IgG, IgM) in human B-cell culture supernatants were measured using commercially available ELISA kits (ELK Biotechnology, ELK1390, ELK10232, Wuhan, China). Briefly, 96-well plates were coated with capture antibodies, incubated with samples, and then probed with biotin-conjugated detection antibodies. Absorbance was measured at 450 nm, and concentrations were calculated using standard curves.

### 2.12. Statistical Analysis

All statistical analyses were performed using SPSS 23.0 (IBM Corp., San Jose, CA, USA) and GraphPad Prism 7.0 (GraphPad Software, Inc., San Diego, CA, USA). Data are presented as mean ± SEM. Differences between the two groups were assessed using the unpaired Student’s *t*-test. Correlations between continuous variables were analyzed using Spearman’s rank correlation coefficient. A two-tailed *p* < 0.05 was considered statistically significant.

## 3. Results

### 3.1. Transcriptome Analysis Reveals Elevated ZBP1 Expression in SLE Peripheral Blood and B Cells

**Figure 1:**
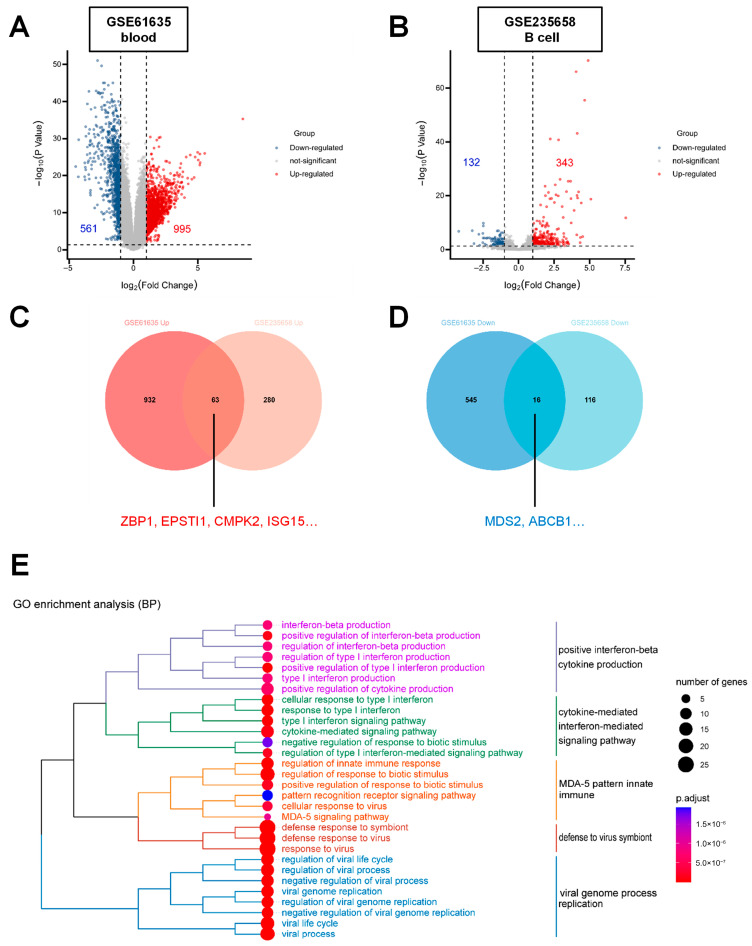
**Transcriptome analysis reveals elevated expression of ZBP1 in peripheral blood and peripheral blood B cells of patients with SLE.** (**A**) Volcano plot showing differentially expressed genes (DEGs) in peripheral blood samples from SLE patients and healthy controls based on the GSE61635 dataset (995 upregulated genes and 561 downregulated genes, |log_2_FC| > 1). (**B**) Volcano plot showing DEGs in peripheral blood B cells from SLE patients and healthy controls based on the GSE235658 dataset (343 upregulated genes and 132 downregulated genes, |log_2_FC| > 1). (**C**) Venn diagram showing 63 commonly upregulated genes shared between the two datasets. (**D**) Venn diagram showing 16 commonly downregulated genes shared between the two datasets. (**E**) Gene Ontology (GO) biological process (BP) enrichment analysis of the 63 commonly upregulated genes, indicating significant enrichment in biological processes related to viral infection, type I interferon signaling, and cytokine-mediated signaling pathways.

To identify key molecular factors involved in SLE, transcriptomic data from two independent GEO datasets (GSE61635: peripheral blood; GSE235658: peripheral blood B cells) were analyzed. In GSE61635, 995 genes were upregulated and 561 genes were downregulated, while in GSE235658, 343 genes were upregulated and 132 genes were downregulated (|log_2_FC| ≥ 1). Among the differentially expressed genes (DEGs), *ZBP1* was significantly increased in both datasets (Figure 1A,B). Venn analysis identified 63 common upregulated and 16 common downregulated genes shared between the two cohorts (Figure 1C,D) (Table 4). GO BP enrichment of the 63 upregulated genes revealed significant associations with viral infection, type I interferon signaling, and cytokine-mediated signaling pathways (Figure 1E), suggesting that *ZBP1* may be linked to interferon-related immune activation in SLE.

To evaluate disease specificity, we additionally examined *ZBP1* expression in B cells from patients with RA using GSE99006 and from pSS using GSE199868. In both autoimmune disease cohorts, ZBP1 expression did not show notable changes relative to healthy controls, supporting that the upregulation of ZBP1 observed in SLE reflects a disease-specific transcriptional feature rather than a generalized autoimmune signature (Supplementary Figure S2).

### 3.2. Single-Cell Transcriptomic Analysis Reveals Upregulation of ZBP1 Across Distinct B-Cell Subsets in SLE

**Figure 2:**
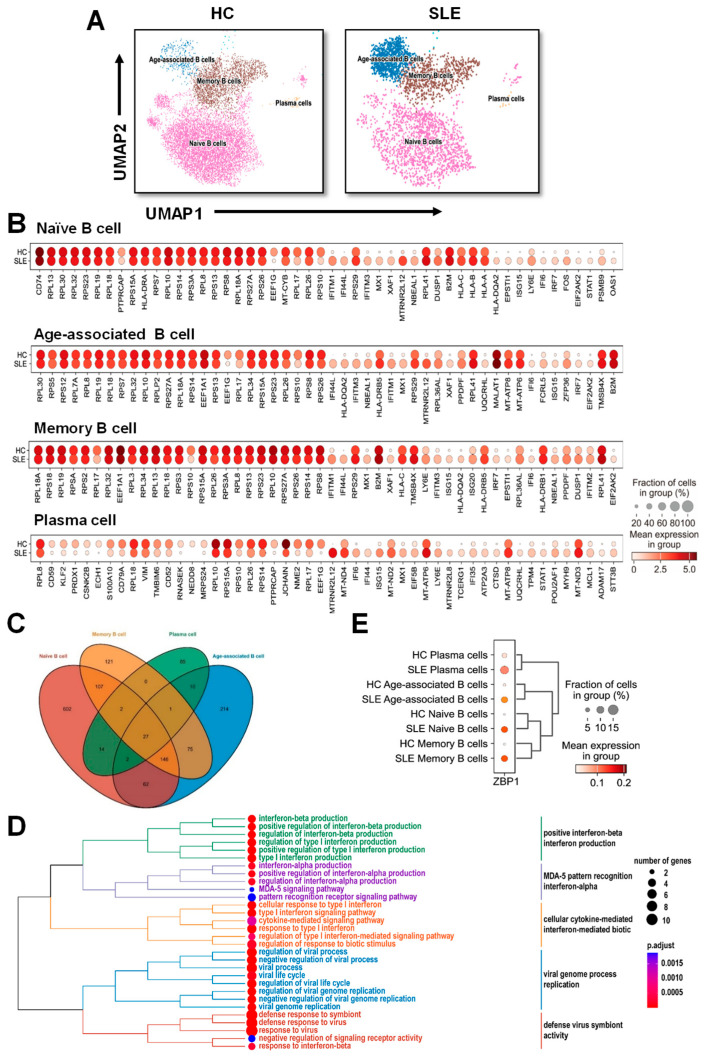
**Single-cell analysis reveals upregulated expression of *ZBP1* in peripheral blood B cell subsets from patients with SLE (GSE136035).** (**A**) UMAP plot showing the clustering of peripheral blood B cells into four major subsets: naïve B cells, age-associated B cells (ABCs), memory B cells, and plasma cells. (**B**) Dot plot showing the top 20 differentially expressed genes (DEGs) across the four B cell subsets between SLE patients and healthy controls. (**C**) Venn diagram showing the overlap of DEGs among the four B cell subsets. (**D**) GO biological process enrichment analysis indicating that the DEGs in these subsets are mainly associated with viral infection, type I interferon signaling, and cytokine-mediated signaling pathways. (**E**) *ZBP1* expression levels are increased in naïve B cells, age-associated B cells, memory B cells, and plasma cells from SLE patients compared with healthy controls.

To further investigate the cell type-specific expression of ZBP1, we analyzed the single-cell RNA sequencing dataset GSE136035, which includes peripheral blood B cells from SLE patients and HCs. After quality control and dimensionality reduction, UMAP visualization revealed clear segregation of peripheral blood B cells into four major subsets: naïve B cells, ABCs, memory B cells, and plasma cells (Figure 2A).

Differential expression analysis identified the top 20 genes that were altered across these B-cell subsets between SLE patients and HCs (Figure 2B). Venn diagram analysis highlighted the overlap of differentially expressed genes among the four subsets, suggesting shared transcriptional alterations associated with disease (Figure 2C). Functional enrichment of these DEGs by GO biological process analysis revealed significant associations with viral infection, type I interferon signaling, and cytokine-mediated signaling pathways (Figure 2D).

Examination of *ZBP1* expression showed consistent upregulation in all four B-cell subsets from SLE patients compared with HCs, including naïve B cells, ABCs, memory B cells, and plasma cells (Figure 2E). These results indicate that *ZBP1* elevation is a general feature of dysregulated B cells in SLE and may be linked to interferon-mediated immune activation across multiple stages of B-cell differentiation.

### 3.3. Transcriptomic and Flow Cytometric Validation Confirm Upregulation of ZBP1 in SLE B Cells and Its Association with Disease Activity

**Figure 3:**
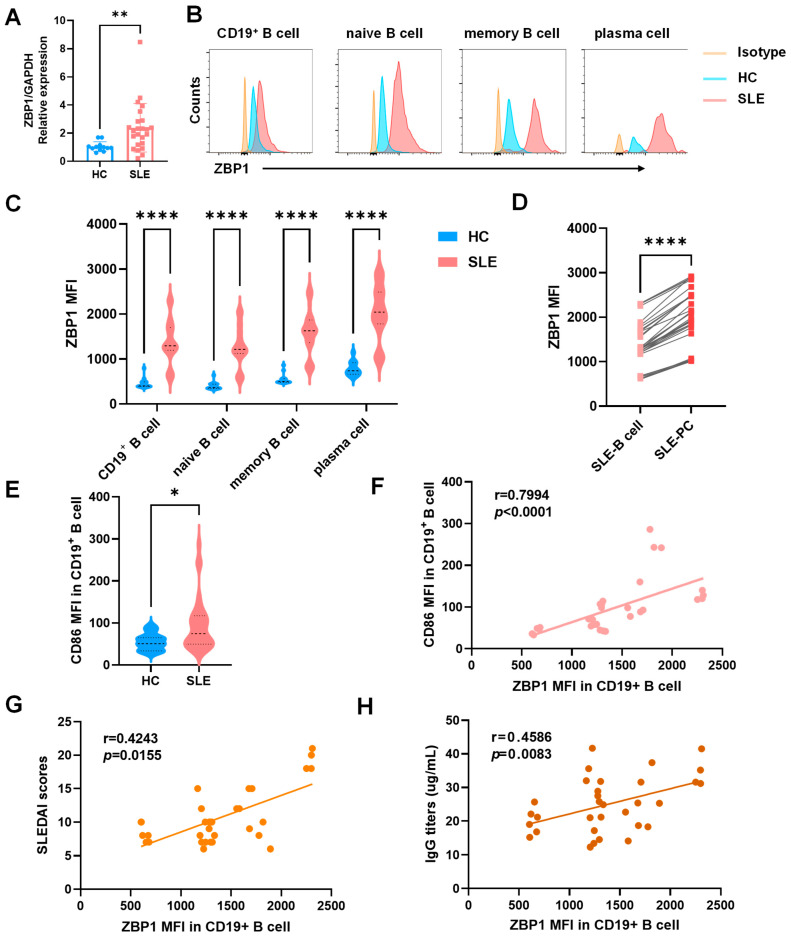
**ZBP1 is upregulated in peripheral blood B-cell subsets from patients with SLE and correlates with B-cell activation and disease activity.** (**A**) RT–qPCR validation showing significantly increased *ZBP1* mRNA expression in purified CD19^+^ B cells from SLE patients (*n* = 24) compared with healthy controls (*n* = 12). (**B**) Representative flow cytometry plots showing increased ZBP1 protein expression in total CD19^+^ B cells and in naïve, memory, and plasma cell subsets from SLE patients. (SLE *n* = 32, HC *n* = 12) (**C**) Quantification of ZBP1 mean fluorescence intensity (MFI) across B-cell subsets. (**D**) ZBP1 expression was further elevated in plasma cells relative to CD19^+^ B cells in SLE patients. (**E**) The activation marker CD86 exhibited elevated MFI in CD19^+^ B cells from SLE patients. (**F**) Positive correlation between ZBP1 MFI and CD86 MFI (r = 0.7994, *p* < 0.0001). (**G**) Correlation between ZBP1 MFI and SLEDAI score (r = 0.4243, *p* = 0.0155). (**H**) Correlation between ZBP1 MFI and serum IgG levels (r = 0.4586, *p* = 0.0083). Data are presented as mean ± SEM. Statistical analyses were performed using the Mann–Whitney U test for group comparisons and Spearman’s correlation for association analyses. *, *p* < 0.05; **, *p* < 0.01; ****, *p* < 0.0001.

To validate the transcriptomic findings, we first assessed *ZBP1* mRNA levels in purified CD19^+^ B cells from SLE patients (*n* = 24) and HCs (*n* = 12). RT-qPCR analysis demonstrated a significant increase in *ZBP1* transcript expression in SLE B cells, consistent with the RNA-seq-based observations (Figure 3A).

We next examined ZBP1 protein expression in PBMCs from SLE patients (*n* = 32) and HCs (*n* = 12) using flow cytometry. As shown in Figure 3B,C, ZBP1 expression was significantly increased in total CD19^+^ B cells (HC, 462.83 ± 34.72; SLE, 1384.31 ± 88.85), as well as in naïve (HC, 405.42 ± 25.15; SLE, 1281.9 ± 80.29), memory (HC, 557.58 ± 35.85; SLE, 1601.53 ± 88.23), and plasma (HC, 784.92 ± 46.13; SLE, 2034.69 ± 108.21) B-cell subsets from SLE patients compared with healthy controls. Moreover, ZBP1 expression was highest in plasma cells (PCs), suggesting its association with terminal differentiation (Figure 3D). The activation marker CD86 was also elevated in SLE B cells (Figure 3E). Correlation analyses revealed that ZBP1 MFI was positively correlated with CD86 MFI (r = 0.7994, *p* < 0.0001; Figure 3F), SLEDAI score (r = 0.4243, *p* = 0.0155; Figure 3G), and serum IgG titers (r = 0.4586, *p* = 0.0083; Figure 3H). Additional serological analyses demonstrated that ZBP1 expression also showed positive correlations with anti-C1q antibody titers and anti-ribosomal P protein antibodies, whereas no significant association was observed with anti-dsDNA autoantibodies. These expanded correlations are presented in Supplementary Figure S3 and further support the clinical relevance of ZBP1 expression in SLE. Collectively, these data provide consistent transcriptional and protein-level evidence that ZBP1 is upregulated in SLE B cells and closely linked to B-cell activation and clinical disease activity.

### 3.4. Transcriptomic Profiling After ZBP1 Knockdown Reveals Enrichment of Cell Cycle and p53 Signaling Pathways

**Figure 4:**
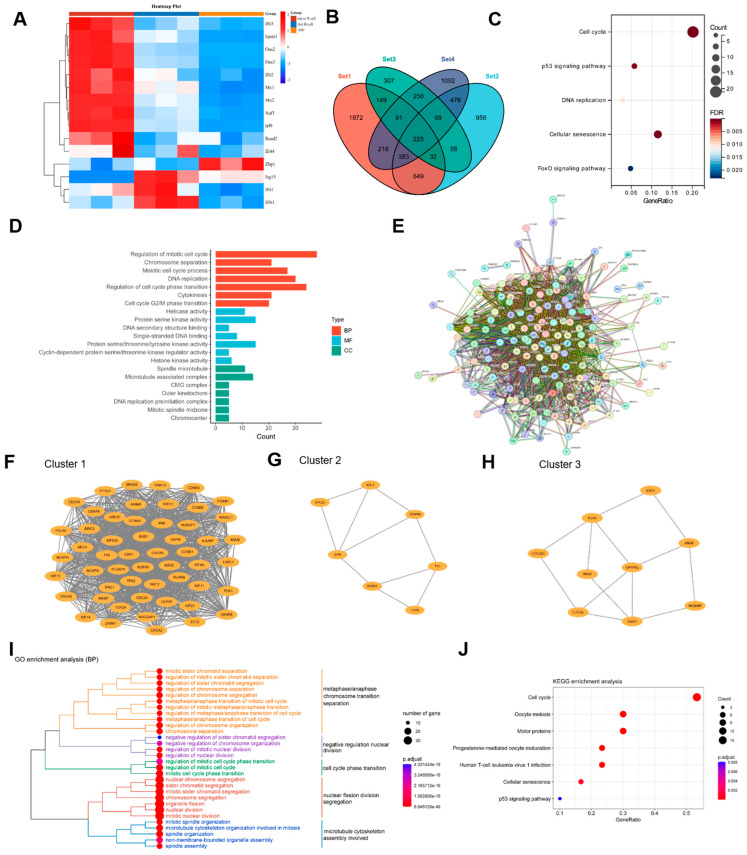
**Transcriptomic profiling after ZBP1 knockdown reveals enrichment of cell cycle and p53 signaling pathways.** (**A**) Analysis of mouse B-cell subset RNA-seq data showed that Zbp1 expression is markedly upregulated in antibody-secreting cells (ASCs) compared with naïve and activated B cells. (**B**) Venn diagram displaying overlapping differentially expressed genes (DEGs) from the RNA-seq dataset GSE163497 following ZBP1 knockdown in multiple myeloma (MM) cells using different shRNA constructs. (**C**) KEGG pathway analysis of overlapping DEGs revealed significant enrichment in pathways related to cell cycle regulation, p53 signaling, and DNA replication. (**D**) GO enrichment analysis demonstrated that DEGs were enriched in biological processes (BP) associated with mitotic cell cycle progression, DNA replication, and G2/M phase transition; molecular functions (MF) including helicase activity and protein serine/threonine kinase activity; and cellular components (CC) associated with DNA replication machinery. (**E**) Protein–protein interaction (PPI) network constructed from overlapping DEGs using STRING. (**F**–**H**) Functional clusters identified within the PPI network using MCODE. (**I**,**J**) GO-BP and KEGG enrichment analyses of Cluster 1 genes showing predominant involvement in cell cycle regulation.

To provide additional support for the relevance of ZBP1 in B-cell terminal differentiation, we first examined our previous sequencing data of mouse B-cell subsets [47], including naïve B cells, activated B cells, and antibody-secreting cells (ASCs). We observed a pronounced upregulation of ZBP1 specifically in ASCs (Figure 4A), suggesting that ZBP1 expression is associated with terminal B-cell differentiation and may contribute to antibody production.

To gain preliminary insight into the downstream molecular programs potentially regulated by ZBP1, we analyzed the RNA-seq dataset GSE163497, which profiled MM cells after ZBP1 knockdown using different shRNA constructs. Volcano plots (Supplementary Figure S4A–D) revealed extensive transcriptional changes across the different shRNA conditions, and Venn analysis identified overlapping DEGs among the treatments (Figure 4B). KEGG pathway analysis indicated that ZBP1-regulated genes were predominantly enriched in cell cycle regulation, p53 signaling, and DNA replication (Figure 4C). GO analysis further revealed enrichment of BP related to mitotic cell cycle, DNA replication, and G2/M phase transition; MF related to helicase activity and protein serine/threonine kinase activity; and CC associated with DNA replication machinery (Figure 4D).

A PPI network constructed from the DEGs (Figure 4E) revealed three major functional clusters via MCODE analysis (Figure 4F–H), with Cluster 1 significantly enriched in genes related to cell cycle regulation, as confirmed by GO-BP and KEGG analyses (Figure 4I,J). Collectively, these results suggest that ZBP1 may influence cellular proliferation and activation programs, providing preliminary mechanistic insight into its potential role in B-cell hyperactivation in SLE.

### 3.5. ZBP1 Knockdown Impairs B-Cell Activation, Plasma Cell Differentiation, and Antibody Production In Vitro

**Figure 5:**
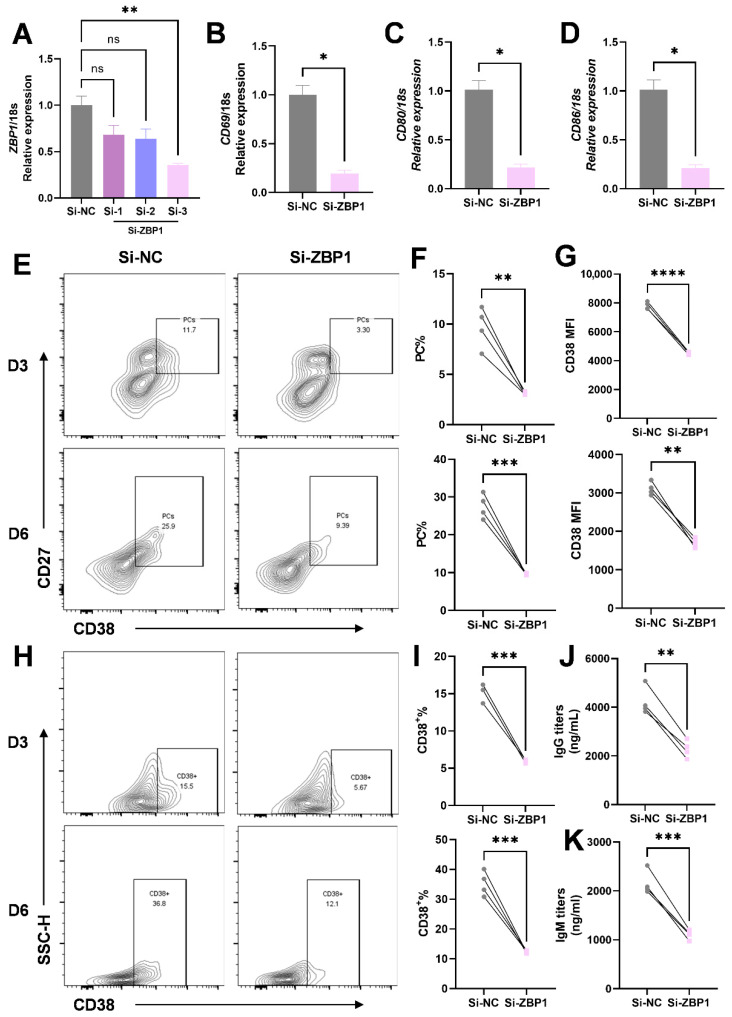
**ZBP1 promotes B-cell differentiation and antibody production.** (**A**) Validation of siRNA-mediated ZBP1 knockdown efficiency and selection of the most effective siRNA fragment. (**B**–**D**) mRNA expression levels of B-cell activation markers CD69, CD80, and CD86 in Raji B cells after transfection with si-ZBP1 or negative control siRNA (si-NC). (**E**) Representative flow cytometry plots showing the proportion of plasma cells (PCs) at day 3 (D3) and day 6 (D6) following si-NC or si-ZBP1 treatment. (**F**) Quantification of PC frequencies at D3 and D6, showing a significant reduction after ZBP1 knockdown. (**G**) Statistical analysis of CD38 mean fluorescence intensity (MFI) at D3 and D6, demonstrating decreased CD38 expression upon ZBP1 silencing. (**H**) Representative flow cytometry plots showing the proportion of CD38^+^ cells at D3 and D6 after si-NC or si-ZBP1 treatment. (**I**) Quantification of CD38^+^ cell frequencies at D3 and D6, indicating a significant decrease following ZBP1 knockdown. (**J**,**K**) IgG and IgM titers in culture supernatants at D6, showing reduced antibody production after ZBP1 silencing. *n* = 4. Data are presented as mean ± SEM. Statistical significance was determined using a paired Student’s *t* test. *, *p* < 0.05; **, *p* < 0.01; ***, *p* < 0.001; ****, *p* < 0.0001; ns, not significant.

To investigate the functional role of ZBP1 in B-cell activation, differentiation, and antibody production, we performed siRNA-mediated knockdown of *ZBP1* in Raji B cells. Efficient *ZBP1* silencing was confirmed, and the most effective siRNA fragment was selected for subsequent experiments (Figure 5A). Following *ZBP1* knockdown, the mRNA expression levels of key B-cell activation markers, including *CD69*, *CD80*, and *CD86*, were significantly reduced compared with cells transfected with negative control siRNA (Figure 5B–D). We next assessed the impact of ZBP1 silencing on human B-cell differentiation. Flow cytometric analysis revealed a decreased proportion of PCs at both day 3 (D3) and day 6 (D6) after si-*ZBP1* treatment (Figure 5E,F). Consistently, CD38 expression, a marker associated with plasma cell differentiation, was significantly reduced, as reflected by decreased MFI and a lower frequency of CD38^+^ cells at both time points (Figure 5G–I). Finally, functional assessment of antibody secretion demonstrated that IgG and IgM titers in the culture supernatants at D6 were markedly decreased following ZBP1 knockdown (Figure 5J,K). Collectively, these results indicate that ZBP1 contributes to B-cell activation, plasma cell differentiation, and immunoglobulin production in vitro.

## 4. Discussion

In this study, we identified ZBP1 as a novel molecule associated with B-cell dysregulation in SLE. Through integrative transcriptomic analyses, we demonstrated that ZBP1 expression is markedly elevated in the peripheral blood and B cells of SLE patients, and its expression correlates positively with B-cell activation markers and clinical disease activity indices. Additionally, our in vitro experiments further support a functional association between ZBP1 and B-cell responses. Knockdown of *ZBP1* in B cells resulted in attenuated expression of activation markers, impaired plasma cell differentiation, and reduced antibody production. These findings point to a potential role for ZBP1 in promoting abnormal B-cell activation and the breakdown of immune tolerance, two key hallmarks of lupus pathogenesis.

Analysis of two independent bulk transcriptome datasets (GSE61635 and GSE235658) consistently revealed upregulation of ZBP1 in SLE, accompanied by enrichment of pathways related to type I IFN signaling, viral infection, and cytokine-mediated immune responses. These enrichment results place ZBP1 within the IFN-inducible gene network that is central to lupus immunopathology. Chronic activation of the type I IFN pathway represents a major pathogenic axis in SLE, shaping the transcriptional profile and functional state of B cells, T cells, dendritic cells, and monocytes. Among these, B cells are major responders to IFN-α, which promotes their activation, antigen presentation, and differentiation into plasma cells capable of secreting high-affinity autoantibodies [14,17]. Therefore, the persistent overexpression of ZBP1 in lupus B cells may further amplify IFN signaling, leading to sustained B-cell activation and expansion of autoreactive clones.

Our single-cell RNA sequencing analysis (GSE136035) confirmed that ZBP1 expression is broadly elevated across multiple B-cell subsets, including naïve, memory, ABCs, and plasma cells, rather than being restricted to a specific subset. This pattern is consistent with the strong interferon milieu characteristic of SLE and suggests that ZBP1 upregulation in B cells is, at least in part, interferon-driven. Notably, although ZBP1 expression was not exclusive to ABCs, its elevated level in this subset—known to be enriched for autoreactive specificities and linked to autoantibody production—raises the possibility that ZBP1 may contribute to pathogenic B-cell activation in a permissive inflammatory context. Importantly, recent studies have reported that ZBP1 promotes germinal center B-cell responses and enhances antibody production following viral infection [39], supporting a functional role for ZBP1 in amplifying humoral immune responses beyond serving as a passive interferon marker. Given that germinal center reactions and plasma cell differentiation are exaggerated in SLE [50,51,52,53], our findings suggest that ZBP1 may function as an interferon-responsive molecular mediator that links nucleic acid-sensing pathways to heightened B-cell activation. However, whether ZBP1 exerts interferon-independent effects in primary SLE B cells warrants further investigation.

Clinically, we observed that ZBP1 expression correlates positively with CD86 expression, SLEDAI scores, and serum IgG levels, linking it directly to enhanced B-cell activation and disease activity. CD86 is a key costimulatory molecule necessary for B–T cell interaction and T-cell activation [54,55]. Thus, ZBP1 may contribute to the amplification of pathogenic B–T cell crosstalk, further promoting autoantibody generation and tissue inflammation. These findings collectively indicate that ZBP1 overexpression in lupus B cells is not merely a secondary byproduct of IFN signaling but may contribute functionally to the autoimmune cascade.

To investigate the functional consequences of ZBP1 silencing, we examined the ZBP1 knockdown RNA-seq dataset (GSE163497) derived from MM cells. While MM cells differ from normal B cells, their shared lineage characteristics, particularly their plasma cell phenotype, high immunoglobulin production, and proliferative nature, make them a valuable model for studying B-cell-related gene networks [56,57]. In addition, primary human B cells are notoriously difficult to transfect, and thus MM cells offer a technically feasible system to probe transcriptional effects of ZBP1 depletion. Intriguingly, ZBP1 knockdown led to the downregulation of cell cycle-related genes, DNA replication machinery, and components of the p53 signaling pathway, suggesting that ZBP1 contributes not only to innate immune responses but also to the regulation of cell proliferation and survival. These results imply a dual role for ZBP1 in coupling immune activation with proliferative signals that sustain autoreactive B-cell expansion.

Mechanistically, ZBP1 (also known as DAI or DLM-1) is a cytosolic DNA sensor that recognizes Z-form nucleic acids and activates downstream signaling through STING–TBK1–IRF3 and NF-κB pathways [58,59,60,61]. This leads to the induction of type I IFNs and proinflammatory cytokines. Persistent activation of this axis contributes to chronic IFN exposure, a defining molecular signature of SLE. Importantly, IFN-α can further enhance ZBP1 expression in a positive feedback loop, thereby reinforcing IFN-dependent transcriptional programs. Such a self-sustaining IFN–ZBP1 amplification circuit could help explain the chronic immune activation observed in lupus B cells. Furthermore, ZBP1-mediated activation of NF-κB may directly promote the transcription of genes involved in B-cell costimulation, antigen presentation, and cytokine production. Thus, ZBP1 acts as a molecular node linking nucleic acid sensing, IFN signaling, and B-cell activation.

Beyond its canonical role in antiviral defense, our data suggest that ZBP1 also modulates cell cycle progression and DNA damage responses, as evidenced by the suppression of p53 and cell proliferation pathways after ZBP1 knockdown. These findings raise the possibility that ZBP1 may facilitate the survival and expansion of autoreactive B cells by attenuating checkpoint control mechanisms. Indeed, accumulating evidence indicates that lupus B cells display intrinsic abnormalities in apoptosis, DNA repair, and metabolic reprogramming [17]. ZBP1, by integrating innate sensing and proliferative pathways, could thus serve as a pivotal regulator of B-cell fate decisions in the autoimmune setting.

Taken together, our study provides new insights into the potential role of ZBP1 in SLE B cells. While ZBP1 is a classical interferon-stimulated gene, we systematically profiled its expression across multiple B-cell subsets, including naïve, memory, ABCs, and plasma cells, using both bulk and single-cell transcriptomic analyses, revealing subset-specific upregulation not previously reported. Clinically, ZBP1 expression in peripheral B cells correlates with indices of B-cell activation (CD86 MFI), disease activity (SLEDAI), and serum IgG levels, suggesting a link to functional B-cell status beyond IFN signaling alone. RNA-seq analysis following ZBP1 knockdown in multiple myeloma cells highlights candidate downstream pathways, cell cycle regulation, DNA replication, and p53 signaling, that may distinguish ZBP1 from other canonical ISGs in influencing B-cell proliferation and terminal differentiation. Furthermore, in our previous sequencing data of mouse B-cell subsets, ZBP1 was specifically upregulated in antibody-secreting cells, supporting its potential role in B-cell terminal differentiation and antibody production. Collectively, these findings support a model in which ZBP1 overexpression in lupus B cells amplifies IFN-driven activation, promotes abnormal differentiation, and contributes to pathogenic expansion, potentially linking nucleic acid sensing to B-cell hyperactivation and autoantibody production.

Despite these insights, several limitations and considerations regarding the novelty of the study should be acknowledged. First, the publicly available transcriptomic datasets analyzed here lack sufficient clinical metadata. The bulk RNA-seq datasets (GSE61635 and GSE235658) do not provide information on disease activity, treatments, or organ involvement, and the single-cell dataset (GSE136035) includes only three SLE patients without detailed annotation. As a result, cohort comparability could not be assessed, and stratified analyses based on clinical features were not feasible. Second, although ZBP1 knockdown experiments revealed functional effects on B-cell activation, plasma cell differentiation, and antibody secretion in vitro, definitive mechanistic validation in primary B cells from SLE patients remains limited. In this regard, we acknowledge that the use of multiple myeloma cells for ZBP1 knockdown provides only preliminary mechanistic clues, as these terminally differentiated malignant plasma cells cannot fully represent the biology of naïve, memory, or autoreactive B cells. Nevertheless, because both SLE and multiple myeloma share features of exaggerated plasmablast/plasma cell differentiation and heightened immunoglobulin production, these results offer a useful initial reference point and generate testable hypotheses for future studies. Third, although ZBP1 is a well-established interferon-stimulated gene, its specific involvement in human SLE B-cell dysregulation has not been previously characterized. In this study, we not only integrated multi-cohort transcriptomic datasets but also validated ZBP1 overexpression at both the protein and mRNA levels in B cells from an independent SLE cohort, thereby providing new evidence for its relevance in SLE pathogenesis. Fourth, the upstream drivers of ZBP1 induction in SLE remain undefined; cytosolic DNA accumulation, mitochondrial stress, and impaired nucleic acid clearance, all common in lupus, may contribute but require direct experimental investigation. Furthermore, in vivo studies using lupus-prone mouse models (such as MRL/lpr or NZB/W F1) will be essential for confirming the pathogenic importance of ZBP1 and evaluating its potential as a therapeutic target in systemic autoimmunity Importantly, the present study did not specifically assess ZBP1 expression or function in regulatory B-cell (Breg) subsets, which are increasingly recognized as critical modulators of immune tolerance and are known to be functionally impaired in SLE. Our study primarily focused on conventional B-cell populations associated with activation, differentiation, and antibody production, and therefore the potential role of ZBP1 in regulatory B-cell compartments was not addressed. As a result, whether ZBP1 contributes to SLE pathogenesis not only by promoting pathogenic B-cell activation but also by modulating Breg-mediated immune regulation remains unclear. This represents an important direction for future investigation, particularly in light of recent studies highlighting the pivotal role of Bregs in SLE. Finally, an important limitation of this study is that the precise contribution of interferon signaling to ZBP1-mediated B-cell activation has not been fully resolved. While ZBP1 is an interferon-inducible gene, existing evidence suggests that it may also participate in downstream signaling processes that amplify adaptive immune responses. Dissecting interferon-dependent and -independent mechanisms of ZBP1 action will be an important focus of future studies.

## 5. Conclusions

In conclusion, our study identifies ZBP1 as a previously unrecognized regulator of B-cell activation in SLE. By integrating innate nucleic acid sensing with IFN signaling and cell cycle control, ZBP1 may promote the expansion and persistence of autoreactive B cells. These findings not only expand the understanding of lupus immunopathogenesis but also suggest that targeting ZBP1 may represent a promising therapeutic strategy for restoring B-cell immune tolerance in SLE.

## Figures and Tables

**Table 1:** Basic information of the two microarrays.

	Subjects	Platform	Organization Name	Country	Submission Date/Updated Date	Number of Samples (SLE/HC)	Reference
GSE61635	Blood	GPL570	Eli Lilly and Company	USA	2014.09.22/2019.03.25	79/30	-
GSE235658	B cells	GPL16791	Emory University	USA	2023.06.23/2024.03.13	8/7	[38]

**Table 2:** The clinical parameters of SLE patients and HCs.

	HC (*n* = 12)	SLE (*n* = 32)	*p* Value
Age	32.17 ± 10.59	31.38 ± 10.23	0.9636
Gender (male/female)	1/11	3/29	>0.9999
SLEDAI	-	10.63 ± 4.14	-
Disease course (months)	-	8.47 ± 11.96	-
Fever, %	-	13/32 (40.63%)	-
Joint swelling and pain, %	-	23/32 (71.88%)	-
Facial erythema, %	-	16/32 (50%)	-
Mouth ulcers, %	-	10/32 (31.25%)	-
Hair loss, %	-	14/32 (43.75%)	-
Fatigue, %	-	12/32 (37.5%)	-
Photosensitivity, %	-	9/32 (28.13%)	-
Dry mouth, %	-	7/32 (21.88%)	-
Dry eyes, %	-	7/32 (21.88%)	-
Raynaud phenomenon, %	-	10/32 (31.25%)	-
WBC (×10^9^/L)	-	4.1 ± 2.31	-
PLT (×10^9^/L)	-	196.41 ± 90.33	-
Hb (g/L)	-	102.91 ± 22.06	-
N (×10^9^/L)	-	3.28 ± 2.2	-
L (×10^9^/L)	-	0.83 ± 0.72	-
NLR	-	4.98 ± 2.45	-
ALB (g/L)	-	32.7 ± 6.11	-
GLB (g/L)	-	36.5 ± 7.17	-
A/G	-	0.93 ± 0.31	-
ALT (U/L)	-	26.62 ± 19.51	-
AST (U/L)	-	34.42 ± 18.4	-
BUN (mmol/L)	-	4.68 ± 1.9	-
Cr (μmol/L)	-	63.34 ± 12.39	-
ESR (mm/h)	-	85.41 ± 31.82	-
CRP (mg/L)	-	12.62 ± 23.86	-
IgG (g/L)	-	25.03 ± 8.22	-
IgM (mg/L)	-	1654.5 ± 808.41	-
IgA (mg/L)	-	5872.34 ± 7097.22	-
C3 (mg/L)	-	305.6 ± 213.74	-
C4 (mg/L)	-	108.72 ± 69.1	-
Urine protein	-	1.22 ± 1.72	-
Anti C1q antibody (U/mL)	-	11.08 ± 6.65	-
Antinuclear antibody	-	1:160 (2/32) 1:320 (30/32)	-
Anti-dsDNA antibody, %	-	26/32 (81.25%)	-
Anti-nRNP/Sm antibody, %	-	22/32 (68.75%)	-
Anti-Sm antibody, %	-	19/32 (59.38%)	-
Anti-SSA antibody, %	-	23/32 (71.88%)	-
Anti-RO52 antibody, %	-	19/32 (59.38%)	-
Anti-SSB antibody, %	-	13/32 (40.63%)	-
Anti-Scl-70 antibody, %	-	2/32 (6.25%)	-
Anti-centromere antibody, %	-	2/32 (6.25%)	-
Anti-nucleosome antibody, %	-	17/32 (53.13%)	-
Anti-histone antibody, %	-	16/32 (50%)	-
Anti-ribosomal P antibody, %	-	17/32 (53.13%)	-

Notes: All clinical laboratory parameters were measured in the Clinical Laboratory of Xiangya Hospital using standardized diagnostic assays. Autoantibodies (ANA profile, anti-dsDNA, anti-Sm, anti-RNP, anti-SSA, anti-SSB, anti-C1q, anti-ribosomal P protein, etc.) were detected using a commercial immunoblot strip assay. Autoantibody positivity (%) indicates the proportion of patients within the SLE cohort who tested positive for each specific autoantibody.

**Table 3:** The primers used in this study for RT-qPCR.

Primer	Forward	Reverse
*GAPDH*	CAGGAGGCATTGCTGATGAT	GAAGGCTGGGGCTCATTT
*18S*	GTAACCCGTTGAACCCCATT	CCATCCAATCGGTAGTAGCG
*ZBP1*	GACTTGAGCACAGGAGACAATCTGG	CTTGGGCACTTGGCATTTCTTCAC
*CD69*	CAGACATGGAAATGGGCAAATGGC	CCTCACAGTCCACAGCGGTAAC
*CD80*	CTCTTGGTGCTGGCTGGTCTTTC	AGGACAGCGTTGCCACTTCTTTC
*CD86*	TCTGCCGTGCCCATTTACAAAGG	TGTGCCCAAATAGTGCTCGTACAG

**Table 4:** Identification of DEGs, including 63 up-regulated genes and 16 down-regulated genes.

DEGs	Gene Name
Up-regulated	*PLSCR1*, *PTP4A1*, *EPSTI1*, *PARP9*, *SAMD9L*, *SCO2*, *ZBP1*, *CSRNP1*, *IRF7*, *OASL*, *FOS*, *SOCS3*, *IFIT3*, *MX2*, *IFI44*, *EIF2AK2*, *ZCCHC2*, *HERC5*, *IFI6*, *IFI44L*, *TRIB1*, *RSAD2*, *SERPING1*, *DDX60L*, *ODF3B*, *OAS3*, *CMPK2*, *ISG15*, *XAF1*, *APOL6*, *NFIL3*, *OAS1*, *IFIT1*, *IFI35*, *STAT1*, *LINC00487*, *LAMP3*, *MX1*, *SPATS2L*, *DDIT3*, *LY6E*, *DDX60*, *CXADR*, *GBP1*, *DHX58*, *HERC6*, *OAS2*, *PMAIP1*, *USP18*, *TYMP*, *OSM*, *SIGLEC1*, *FBXO6*, *GBP5*, *LAP3*, *RNF103*, *BTG3*, *MT2A*, *MACROD2*, *TOP2A*, *IFI27*, *LGALS3BP*, *KLHDC7B*
Down-regulated	*SBF2-AS1*, *LINC01355*, *ZNF540*, *XKR6*, *THNSL1*, *ARHGAP32*, *LPAR5*, *HOOK1*, *MCF2L*, *ABCB1*, *MDS2*, *PASK*, *LINC00494*, *IL23A*, *NT5E*, *CD200*

## Data Availability

All RNA-seq and single-cell RNA-seq data in this study were downloaded from the GEO database, including GSE61635, GSE235658, GSE99006, GSE199868, GSE136035, and GSE163497. Additional raw data generated in this study are available from the corresponding author upon reasonable request.

## Supplementary Materials

The following supporting information can be downloaded at: https://www.mdpi.com/article/10.3390/biomedicines14020451/s1, Figure S1: Analysis of B cell subtypes in SLE patient PBMCs; Figure S2: Analysis of ZBP1 expression in B cells from RA and pSS patients; Figure S3: Additional correlations between ZBP1 expression in SLE B cells and clinical parameters; Figure S4: Transcriptomic analysis after ZBP1 knockdown in MM cells (GSE163497).
