# Supplementary material for: Upregulated ZBP1 Is Associated with B-Cell Dysregulation in Systemic Lupus Erythematosus
Source: Biomedicines. 2026 Feb 17;14(2):451. doi: 10.3390/biomedicines14020451 (PMC12938334; doi:10.3390/biomedicines14020451)
Supplement: Supplementary file 1 [file biomedicines-14-00451-s001.zip › biomedicines-4134411-supplementary.pdf]

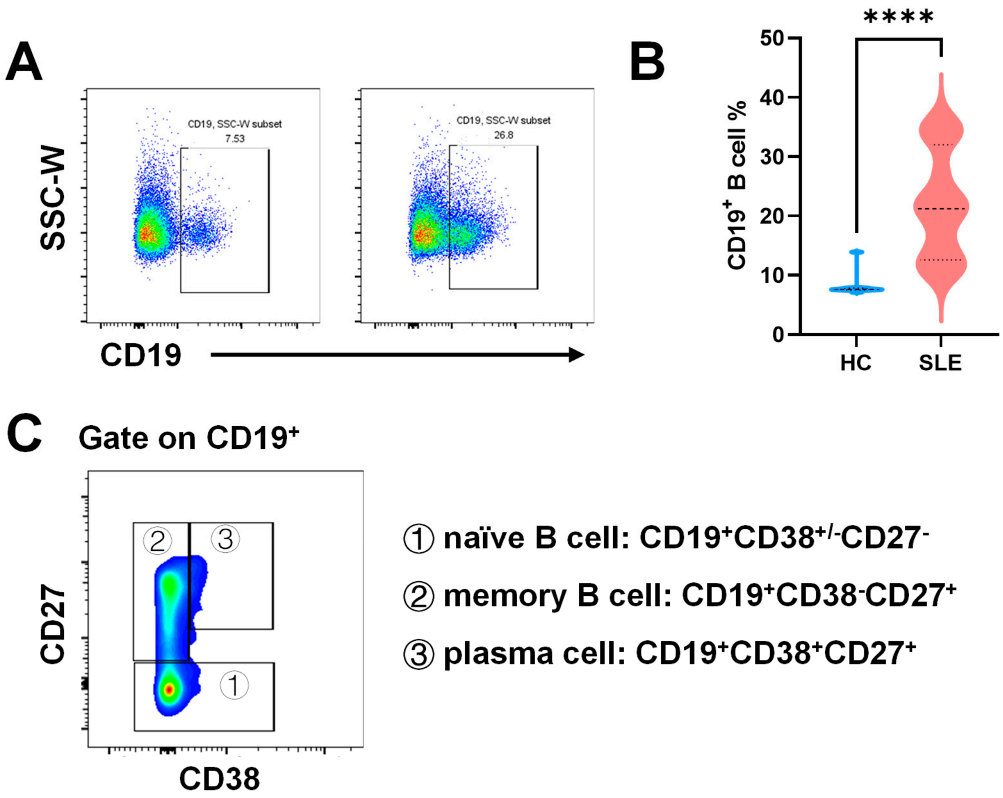

Supplementary Figure S1. Analysis of B cell subtypes in SLE patient PBMCs. (A). Representative flow cytometry plot showing $CD19^+$B cells% in PBMC from SLE patients (n=32) and HCs (n=12). (B). Scatter plot showing $CD19^+$B cells% in PBMCs from SLE patients (n=32) and HCs (n=12). (C). Representative flow cytometry plot showing plasma cells, naive B cells, and memory B cells.

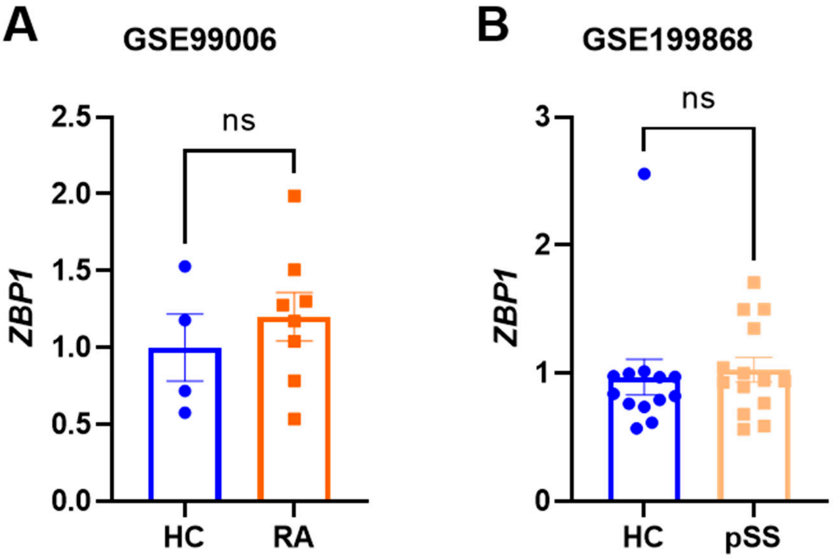

Supplementary Figure S2. Analysis of ZBP1 expression in B cells from RA and pSS patients. ZBP1 expression in B cells from RA patients (A, GSE99006) and from

pSS patients (B, GSE199868) showed no significant differences compared with healthy controls.

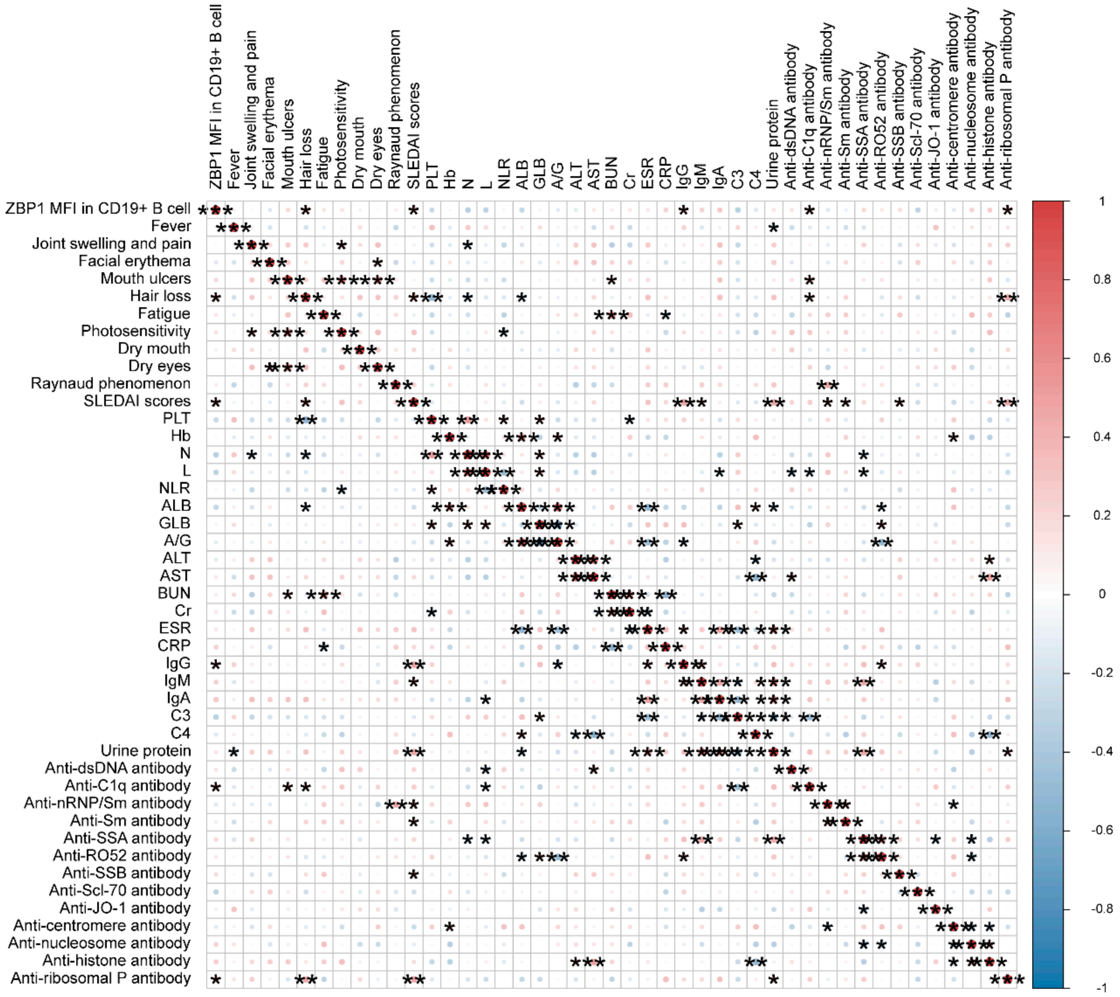

Supplementary Figure S3. Additional correlations between ZBP1 expression in SLE B cells and clinical parameters. Correlation analyses were performed to further evaluate the clinical relevance of ZBP1 expression in CD19^{+} B cells from patients with SLE. ZBP1 mean fluorescence intensity (MFI) showed additional significant associations with multiple clinical indices, including serum anti-C1q antibody titers and anti–ribosomal P protein antibodies, as well as other available laboratory and disease activity parameters. Statistical significance was assessed using Spearman's correlation analysis.

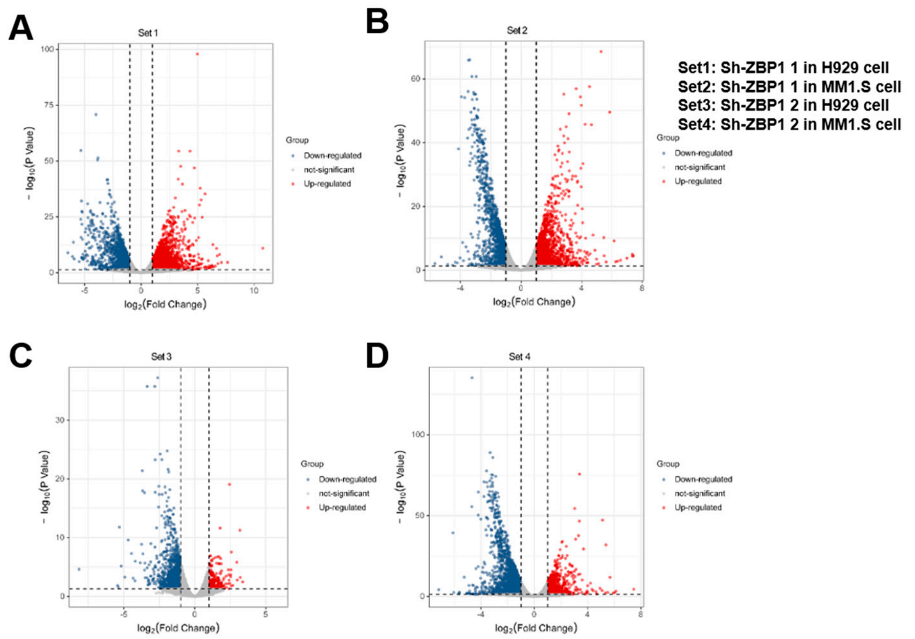

Supplementary Figure S4. Transcriptomic analysis after ZBP1 knockdown in MM cells (GSE163497). (A–D) Volcano plots showing differentially expressed genes (DEGs) in multiple myeloma (MM) cells transfected with different shRNA constructs targeting ZBP1, compared with their respective control groups. Upregulated and downregulated genes are highlighted, illustrating the transcriptional alterations induced by ZBP1 knockdown.
